# Decision making among residents in training of obstetrics and gynecology: A qualitative exploration in Pakistani context

**DOI:** 10.1371/journal.pone.0287592

**Published:** 2023-11-02

**Authors:** Sana Younas, Saeeda Khanum, Azher Hameed Qamar

**Affiliations:** 1 Department of Behavioral Sciences, School of Social Sciences and Humanities, National University of Sciences and Technology, Islamabad, Pakistan; 2 School of Social Work, Lund University, Lund, Sweden; UNITED STATES

## Abstract

Medical decision-making is critical and the decisions are made under uncertain, complex, and dynamic conditions. In this regard, practitioners’ experiences and perceptions may provide a bottom-up knowledge of the issues, as well as a corresponding support system that assists them in learning to make decisions in critical situations. The current study aimed to examine these experiences in the Pakistani context. We interviewed 14 trainee residents (aged 26 to 34 years) from tertiary care hospitals. Using inductive thematic analysis, we explored a participant-centered perspective on the support system and decision-making process. Findings reveal that the major challenges to decision-making include uncertain and complex situations, hospital-related constraints, and sociocultural context. Both non-critical and critical case management are used in individual and group decision-making processes. The residents use knowledge-based, emotional, and instrumental support to make decisions. The study gave practitioners and academics a transdisciplinary platform to explore the cognitive, social, and behavioral aspects of decision-making in the healthcare industry.

## Introduction

Decision-making is central to medical practice because health outcomes are probabilistic. Most medical decisions are made under uncertain, complex, dynamic, and continuously changing conditions [1, 2]. In uncertain and complex situations of health care, it is difficult to involve patient preferences, values, and costs in a complex web of therapeutic and diagnostic uncertainties. Often, there is considerable disagreement about the accurate course of action. Hence, most of the decisions in healthcare are made under conditions of uncertainty. This uncertainty is about patient management that may arise from inaccuracy in the technical recording of clinical findings, erroneous observation, or misinterpretation of the data by the healthcare provider [3]. Furthermore, decision-making in health care becomes complicated because of the factors such as multiple perspectives and backgrounds of the decision-makers, varying interpretations of evidence, and ambiguity of information. These conditions are the characteristics of decision-making that occur daily in healthcare settings [4]. Additionally, decision-making in health care typically requires the integration of complex evidence from a variety of sources. This evidence can be conflicting or it may lead to an ambiguous understanding of a patient’s condition. Medical cases that contain anomalous evidence significantly characterize the role of expert decision-making. The nature of the decision task including its difficulty and the extent to which the task is challenging are the factors that have remained to be explored when considering the decision makers.

In health care settings, there are two categories of decision makers; one is the decisions made by the providers on behalf of the patients and the other is the decisions made by both providers and patients collectively. The second type is commonly referred to as shared decision-making [5]. In this study, we are focusing on the first type i.e., decisions made by providers i.e., doctors in hospital settings. Specifically, our focus is on residents in the training of Obs and Gynae. A resident physician is commonly called a senior resident medical officer or a senior house officer in commonwealth countries. Residents hold a medical degree (MD, MBBS) from an accredited medical school. Depending upon the program and specialty, the duration of residency can range from three years to seven years. In the United States, the first year of residency is known as an internship. In Pakistan, residents are doctors who have passed five years of medical school, one year served as a house officer in a hospital, and have passed the Fellow of College of Physicians and Surgeons exams (FCPS-I). It is also a 4–5 years program depending upon the nature of the field. In the present study, we have included the residents who have passed FCPS I exams in the field of Obs and Gynae and are undergoing four years of post-graduate training in tertiary care hospital.

The core value of our medical training in tertiary care lies in the science, affected by the technology, and conducted in larger tertiary care hospitals that are governed by patriarchal ideology. As far as the Obs and Gynae is concerned, more than 20 years have passed but most routine obstetrical procedures such as induction of labor and cesarian delivery have either little or no scientific evidence for justification. They are routinely performed because they make cultural sense rather than scientific sense [6]. In this connection, a technocratic model of birth views the human body as a machine and an object for medical treatment. Hence, a medical practitioner may become a techno-medical practitioner who is solely engaged with technical aspects of birth and views human body as a machine and an object for medical treatment [7]. The practitioners learn this alienation in medical schools and residency. This is seen as a way to protect themselves without taking any risks and avoiding emotional attachment with the patient. It logically follows that there is no reason to deal with the patient’s emotions at all. Thus, they are free to protect their feelings from the pain of caring too much. Doctors and patients are not involved together to make decisions; instead, healthcare system and doctors take the decisions.

The above passage explains the practice of Obs and Gynae in light of the technocratic model of birth. For obstetrical and gynae-related issues, Pakistani women prefer to visit lady doctors. As a professional, doctors are working on the technocratic model but women working in Obs and Gynae do empathize with female patients because they have the same sex as their patients. They have (i) cultural support, (ii) social support, and (iii)a sense of comfort is there for the patient and her family that she is visiting a lady doctor for Obs and Gynae related issues. Secondly, the men of the family (male members of the family) also want their women to visit only female gynecologists because of cultural sensitivity. It translates into positivity in two ways: (i) First it helps in their better psychological well-being which also improves their coping abilities. (ii) Second, the women in the field of Obs and Gynae also get encouragement because they interact more with women in this field, unlike any other medical field, and gain comfort and confidence.

### The decision-making process and challenges

The research found when individuals gain experience in their field, they do not use the classical approach to decision-making in real-world scenarios [8]. They gain experience, match patterns, and intervene without conscious awareness of making a decision. This led to the development of Naturalistic Decision Making (NDM). In addition to the important influence of experience in decision-making, this framework emphasizes contextual key factors during decision-making. The key factors include the influence of high-pressure situations, limited time, changeability, and uncontrolled environments. The environment often includes an overall culture and team which influences decision-making [2]. NDM has been found useful in understanding decision-making in many professions that require high stakes, complex scenarios, and limited time [9]. In medical research, NDM is used to identify processes and factors related to decision-making in complex and uncertain situations [10, 11].

The Naturalistic Decision-Making theory (NDM) provides a valid stance to understand the process of decision-making in naturalistic, dynamic, complex situations of tertiary care hospitals. In fields combining short clinical-decision making time and high-stakes scenarios, such as during an emergency, or surgery of any complicated case, NDM can be used to make timely and rapid decisions based on prior knowledge, experience, and learning [12]. However, the use of NDM as a conceptual guide for residents’ decision-making research is limited. Due to the similarity in factors found in health care literature and NDM, such as pattern matching, experience, and socio-cultural challenges, NDM may provide an important framework to improve understanding of residents’ decision-making within the Pakistani healthcare system.

### Study background

The healthcare system in Pakistan suffers from a variety of challenges, such as low budget for healthcare, lack of medical staff, and high maternal and fetal mortality rate [13, 14]. Pakistan spends less than 1 percent of its GDP (Gross Domestic Product) on healthcare. The government hospitals are short of beds, nurses, and doctors. The regular staff members are mostly postgraduate trainees (residents) who are unable to independently manage pregnancy-related complications. Tertiary care hospitals located in the urban settings of Pakistan are visited by a large number of patients annually hence the patient influx is so high. The Maternal Mortality Rate (MMR) is 299 per 100,000 live births [15]. Among them, twenty percent of the deaths are caused by maternal complications such as eclampsia (convulsions in a pregnant woman) and post-traumatic hemorrhaging. Moreover, the fetal mortality rate in Pakistan is 56.888 deaths per 1000 live births, [16]. One of the major causes of mortality are unsafe delivery practices and lack of competent doctors. There is inadequate access to emergency obstetric care services and low skilled birth attendance. Only about 40% of deliveries are attended by skilled birth attendants [17]. About 175,000 doctors are registered to serve the population but the high workload, lack of funding, high maternal and fetal mortality rate, poor service structure, and heavy patient influx have serious consequences such as many Pakistani doctors moving abroad for medical practice. Furthermore, some lady doctors stopped the practice due to social compulsions and family issues.

The decision-making process remains questionable in the presence of these shortcomings. The literature focuses on the challenges of the healthcare system, and difficulties in providing care. However, the decision-making process by young medical graduates and trainees has rarely been investigated. The conventional medical curriculum in Pakistan is mostly theory-based learning with less practical exposure to decision-making in complex situations [18]. The present qualitative study explores the mechanism of decision-making in hospitals where residents work under environmental pressure. The study pursued the following research questions.

How do practitioners perceive decision-making in complex situations of obstetrics and gynecology?

- What are the challenges faced by practitioners in decision-making?
- What support system do practitioners use in decision-making?

## Method

The available scales on decision-making are measuring general decision-making styles or approaches (for instance, Melbourne decision-making scale, Flinder decision-making style, etc.) which do not fit into the context of realistic, dynamic settings of Obs and Gynae where a lot of challenges adhere with the physicians’ decision making. Hence, to get a detailed exploration such as participants’ perspectives, experiences, and opinions by interpreting their meanings and actions on the subject, the bottom-up thematic analysis provided us an optimum for comparing the different points of view on the phenomenon of naturalistic decision-making. In general, the qualitative study provides a holistic approach as it comprehends sociocultural values and the individual context analysis of naturalistic decision-making adding meanings to the quantitative number. Further, qualitative study is an interpretative and descriptive approach used to investigate humans in their social world. Bottom-up qualitative research helps to reveal the hidden meanings and the depth of the empirical data by interpretation of perceptions, events, experiences, and processes [19]. In this study, we used qualitative research design with an inductive approach and employed Braun and Clarke’s thematic analysis.

### Sample

Purposive sampling was used to access 14 residents in the training of Obs and Gynae (Age range = 26–34 years, M = 28) who have completed five years of MBBS and passed FCPS I exams. The participants are working as trainee residents in tertiary care teaching hospitals in Islamabad and Rawalpindi. We chose tertiary centers because they are generally the teaching hospitals that provide internships to young physicians after completing medical school, and they have residency training opportunities in different disciplines like peads, surgery, urology, gynae, dermatology, etc. As our sample was residents in training, so tertiary care hospitals provided us the opportunity to collect data. The choice of the center can have an impact on the data generation because majority of the specialist doctors can be found at tertiary care. Residents can only be approached at tertiary care centers where they are conveniently available and are given training in their particular chosen field. Tertiary care hospitals are both public and private. The public sector tertiary care is open for the general public twenty-four hours without charging a doctor fee whereas the private sector hospitals charge a fee. Hence, the general public from urban as well as rural areas who cannot afford the huge bills of the private sector visit tertiary care hospitals of the public sector.

Fifty percent of the participants have 1–2 years of residency experience, and others have 3–4 years of experience. All participants are women. In Pakistan, women choose the Obs and gynae field, and it is predominantly a women’s profession. The patients also visit women practitioners. Pakistani social and cultural context does not provide space for men to choose gynae as a major professional field, though there are few male gynecologists in Pakistan.

### Data collection procedure

The Head the of Department in each hospital was contacted to take permission for data collection. The data was collected from July 2022–September 2022. It was a post-Covid time in Pakistan when the patients as well as the doctors were not practicing strict Covid routines like wearing masks, sanitizing hands, or keeping a certain distance from one another. Hence, Covid-19 did not impact the data collection process or responses.

After seeking informed consent, we interviewed the participants. All the interviews were audio-recorded. The average time of the interview was 40–45 minutes. The semi-structured interview guide was based on a relevant literature review and the reflection based on the current situation in Pakistani tertiary care hospitals (as observed by the first author during visits to different hospitals and interaction with residents). The guide was revised after taking one mock interview and two pilot interviews with the participants. One participant was a colleague in the medicine department of a public sector hospital while the other was from Obs and Gynae ward. The final interview guide consisted of twenty questions inquiring about the decision-making process, support, and challenges faced by residents (see S1 Appendix). Ethical approval was obtained from the institutional review board of the department of behavioral sciences, school of social sciences and humanities, NUST (Ref. No. 0988/Ethic/01/S3H/070/DBS). In pre-interview meetings we obtained written and verbal informed consent from the participants, providing them with the consent form and information about research aims and objectives, approximate time in taking the interview, the right to withdraw, the value of honest responses, an audio recording of the interview while ensuring safe storage and anonymous reporting of data.

### Process of analysis

For data analysis, we used six steps of reflexive thematic analysis [20] that includes the back-and-forth examination of the data, coding, clustering and inductive analysis. To improve the analytical rigor of the analysis, we used a checklist to examine and strengthen the quality of data analysis (Table 1). We analyzed the transcripts to identify and discuss the common pattern, themes, and underlying structures. The confirmability and dependability were achieved by ensuring the logical flow, procedural rigor, and consistency in interpretation of the data. Transferability (case-specific generalizability) was ensured by the detailed description based on the data’s latent as well as semantic meanings.

**Table 1:** Checklist of thematic analysis.

	Check List	Description
1.	Transcripts	Data detailed transcriptions-cross check of the recoded data
2.	Coding	Equal consideration has been given to all data for coding—data driven coding
3.	Themes	Similarly occurring codes were grouped into an initial theme and are comprehensively grounded in the data. All the themes were cross checked with each other and are reversed checked with the original data. An internal coherence, relationships and interconnections were ensured in the themes and at the same time themes were distinct from each other.
4.	Significant statements	Themes- carefully sorted the relevant significant statements and correspond to the themes.
5.	Analysis	Analysis is not just the description rather interpretation of the data and provides the conceptual depth of the themes. Data analysis connect the research questions of the study with the emergent themes and also provides coherent evidence-based story of the data.
6.	Reporting	Adequate reporting of the research study. Consistency is presented in the research objectives, questions, method and analysis. Researchers are active in the process and analysis showing themes are emerged from the data and are interpreted with reflexive journaling.

## Findings

Considering the depth of the data, we divide the data in three categories:

- Challenges of decision making
- Support system
- Decision-making process

The following section provides description of themes emerged from the data, interpretation, and discussion corresponding above-mentioned categories.

### Challenges faced by residents in decision making

The first category is about the challenges faced by the residents in decision-making. Residents in training were facing unique challenges in complex and dynamic environment of tertiary care hospitals in Pakistan (Table 2).

**Table 2:** Challenges faced by residents in decision making.

Themes	Subthemes
Uncertain and complex situations	Non-adherence of protocols
	Emergency cases
	Time pressure
Hospital related challenges	
	Lack of infrastructure
	Irresponsible behavior
Social and Cultural context	Observance of cultural obligations
	Local knowledge constraints
	Language barrier

### Uncertain and complex situations

Uncertainty in Obs and gynae department was associated with emergency situations that emerge with intricate complexity, high ambiguity, and changing probability. For example, emergency may entail [21] (i) non-stop bleeding (ii) Surgical removal of uterus (iii) Low lying placenta (iv) Fits due to increased blood pressure.

The emergency becomes complex and uncertain because of protocol errors, emergency concern, and time pressure.

#### Non-adherence of protocols

Participants of the study mentioned some errors which occur when doctors do not follow protocols. This can result in severe complications for the patients. In rare cases, when situation becomes complex, the residents shared their concerns regarding protocol errors that happen for three main reasons. First, the senior doctors assigned to the case could not attend in time and there was no substitute. Second, residents miss important monitoring information related to the baby. Third, blindly following the diagnostic reports without follow-up examination. One resident reported,

“Once a patient who was already examined by the consultant came. Her blood pressure was not checked before surgery. She started getting fits after the delivery and suffered from eclampsia. She was immediately shifted to ICU (Intensive Care Unit). Additionally, another patient came after being examined by a consultant. Her reports revealed that the baby’s position is breech. We also showed laziness as we did not do her scan before the cesarian and found cephalic position of baby when operated”

(p. 8)

The participant shared the information regarding mistakes in following the protocols. As before cesarian of any patient, it is necessary to examine the patient’s medical history as well as physical condition. For instance, detailed examination of patient’s BP, weight, history of any allergy, any previous operation, or any other medical condition like diabetes is determined before the cesarian. Sometimes residents forgot to follow the complete protocols before cesarian which results in eclampsia (a condition in which patient gets fits because of high blood pressure). Eclampsia can lead to brain injury and lifelong abnormality for the patient. Additionally, the uncertainty is increased when a specialist doctor misdiagnoses a patient and trainee residents believe in the details provided by the doctor. Without questioning the senior’s clinical examination and following them blindly is the biggest mistake residents commit. In pregnancy, the delivery of patient is highly dependent upon the position of baby inside the womb. Residents did not examine the patient themselves relying on the report written by a consultant. When patient was operated, the baby was found in a cephalic position. Here the skills and competency of a consultant can be questioned but at the same time, the negligence of residents is also evident.

#### Emergency cases

Residents define emergency issues that are uncertain, critical, and need immediate care. Emergency cases occur with non-booked patients as well as with booked patients who get regular checkups from their gynecologists. One participant reported the emergency cases in these words,

“In gynae, the critical scenario is the baby whose life is in danger inside the tummy of the mother…. Secondly, the bleeding patient is critical. Yesterday night, during 6–7 hours of surgery, one patient’s bleeding was not getting stopped. Almost transfused more than 25 units of blood components. The mismanaged patient is also a critical one. Birth of triplets 3, 3, 4, 4 babies is also critical when shifted to NICU.”

(p.5)

In this extract, the participant elaborated on four types of scenarios that can be considered emergency issues. First is the baby inside the womb of the mother. Baby needs proper care and nutrients for healthy growth. The safe delivery of the baby is also a critical task for the residents because the baby in distress which is commonly called fetal distress is a poor outcome. This can be associated with various environmental as well as biological factors. The baby’s heart rate should be monitored timely to assess fetal well-being. The second emergency issue is the patient in active bleeding. Sometimes in post-operative surgeries, a damaged blood vessel may subsequently begin to bleed resulting in heavy loss of blood, and transfusion is required immediately. If a patient loses a huge amount of blood, then she not only becomes anemic but also her chances of survival are at stake. The third is the mismanaged patient. This mismanagement can be because of two reasons (i) the patient not being given immediate care at the time of delivery (ii) non-following of emergency protocols i.e., patient’s examination before cesarian has not been done. The fourth emergency issue is the safe delivery of triplets and quadruplets. This becomes critical if not given immediate neonatal intensive care (NICU).

#### Time pressure

From the residents’ point of view, time pressure is an additional constraint in the complex environment of hospitals. It adds pressure to the ability of decision-making as residents have to respond to emergencies actively and quickly. It also affects their ability to cope at different levels of task complexity. For instance, some reported issues that involve time pressure are (i) eclampsia (a disorder of pregnancy characterized by seizures) and (ii) women with a ruptured uterus. The resident elaborated on this in these words,

“Yesterday night, there was a case of a ruptured uterus for which we cannot wait for the labs because with a ruptured uterus patient goes in shock. We not only have to save the baby, but we have to save the mother also. So, we did an immediate cesarian by shifting her to OT

(p.14)

The participant explained the case of a patient with a ruptured uterus. In this case, residents including senior doctors must decide within minutes whether to do hysterectomy (surgical removal of the uterus) of patient or manage it by applying stitches to ruptured uterus. If the bleeding does not stop even with the application of stitches, then the patient needs an immediate transfusion of blood. Additionally, doctors decide for hysterectomy to save the patient’s life. Here the critical scenario is a decision between life and death. Even a minor delay from doctors can lead to a patient’s death.

### Hospital-related challenges

The condition of services at public sector hospitals is on the downside. According to residents, either there is lack of equipment in the Obs and Gynae ward, or the ones available are torn and in limited quantity. Since public hospitals are open for the public 24 hours without charging a fee but do not provide adequate medical infrastructure to residents and consultants for patient management. This may lead to grave consequences for patients who are compelled to get treatment from public hospitals as they cannot afford the huge bills of private sector hospitals. We divided the challenges into two subthemes.

#### Lack of infrastructure

The physical challenges that ranked topmost include the (i) limited beds in the labor room. (ii) non-availability of medical testing in hospitals and the (iii) usage of non-sterilized instruments. Patient influx is so high that even residents admit two or even three patients against one bed. Residents cannot refuse the admission of any patient either booked or non-booked as tertiary care hospitals provide emergency services to every individual in Pakistan, otherwise, they would be held accountable for the patient’s refusal of admission.

“Limitations come in beds. e.g., we have an accommodation of twenty-five beds in the labor room but if patients are outnumbered then we try to accommodate them in a side room or any place. Other than this, if we see facility-wise; if any test is necessary to be done e.g., beta HCG is to be tested for ectopic pregnancy…. then it is a constraint because its facility is not available in the hospital”.

(p.3)

A participant explains the condition of public sector hospitals that there are limited beds. As residents cannot refuse admission of any patient in tertiary care because of accountability so they must admit the patients in any case. The treatment of any disease is the same in every hospital despite being public or private, but the issue of beds is only in the public sector. In the labor room, they have accommodation of twenty-five beds only but due to the high patient influx, they must take many more patients. Furthermore, hospitals also do not provide service for free medical tests that have to be conducted from laboratories with payment. These tests include beta HCG to check pregnancy and this also helps to detect ectopic pregnancy (pregnancy in the uterine tube). Patients’ laboratory test from outside requires them to pay from their own pockets.

#### Irresponsible behavior

Irresponsible behavior is divided into two types, i.e., non-availability of doctors and noncooperation of staff. In public hospitals, doctors are permanent employees of the Government, and they cannot be turned over unless accused of any serious penalty. Sometimes, doctors show negligence in terms of late arrival in case of emergency patient management. This can also be unconscious because doctors may have been busy in the management of any other patient that was critical too and hence comes late for the next emergency management. But the consequences that patients must face are long-term and heartbreaking. For instance, one resident reported,

“If he is not present on duty, he is sitting at home and takes some time to reach. Last night, the anaesthetist reached too late. We kept on waiting that he may come……but he arrived too late and, in the end, happened stillbirth of a child.”

(p.5)

A resident reported that sometimes doctors are not present on their duty, either sitting at home or coming late when called for an emergency case. This is the dilemma of public sector hospitals in that they take patients for granted. As mentioned earlier that one of the protocols before a cesarian is the availability of doctors for operating the case. Rarely, it happens that the doctor got late in coming when called in an emergency. The late arrival of a doctor can be attributed to two factors. (i) Either the doctor is busy with the examination of another critical patient (ii) or the doctor got late in coming because of the heavy traffic. Once the anesthetist when called before the cesarian of the patient got late in coming. Cesarian cannot be started without the anesthesia doctor who gives local anesthesia to the patient. Due to the late arrival of the anesthesia doctor, the baby died inside the uterine walls.

### Social and cultural context

This is the third theme that explains the influence of social and cultural context as one of the challenges to cope in decision-making process. It includes the pressure from the attendants of the patients and at the cultural level, it is the local knowledge constraints, observance of essential cultural obligations, and language barrier that play a fundamental role as a challenge in decision-making. Let us describe each subtheme one by one.

#### Observance of essential cultural obligations

This subtheme includes the cultural practices prevalent in Pakistani society. Discussion on pregnancy and pregnancy-associated complications is considered a taboo topic in developing countries like Pakistan. As one resident stated,

“Our 40–60% society is not literate till now. The rest 40 to 50% come from beneficiary who only knows that this is our 9th month and when they feel strong pains, it means the child is ready to be born. Other than this, they do not know anything. They have not undergone an anomaly scan. They don’t know about placenta and its complications.”

(p.5)

Here, the participant reflected on the limited knowledge of our society. Pregnancy is not taken seriously by people but when any complication occurs, they become worried. Obs and Gynae is such an uncertain and unpredictable field that an emergency can happen at any time. The literacy rate of Pakistan is only between 40 to 60%. Majority of the people only know pregnancy has total nine months and the delivery time is near when pains become strong at the end of pregnancy. Our society is unfamiliar with all other essential protocols of pregnancy like fetal checkups, blood tests, anomaly scans at 20 weeks of pregnancy, fetal heart rate assessment, fetal growth, placenta position (that provides oxygen to baby), etc. Sometimes cultural practices like not visiting the doctor may lead to severe complications like high blood pressure (BP) which may cause brain injury in patients.

Furthermore, there are some cultural myths associated with contraception. People believe that using them will permanently cause infertility among women [22]. Moreover, the cultural value of the male child [23] in our society is embedded to the extent that women are ready to conceive for the eighth or ninth time without caring for their health.

“And then attendants…. people do not get counsel. They do not get counsel even after ten…ten children. They do not want to use contraception. First, we want to counsel them for their goodness and second, we have to convince them separately.”

(p.6).

In this part, the participant expressed the stress of counseling the attendants. Even after ten children they do not get counsel and are not ready to use contraception. Doctors counsel the patients for two main reasons, (i) the health of women is more important than conceiving and giving birth, (ii) usage of contraception will allow a reasonable gap among the children. This is better for their health as well as proper attention could be given to each child. Resident trainees counsel the patients and their attendants and put great effort to convince them.

#### Local knowledge constraints

This subtheme reflects the local knowledge of midwives regarding pregnancy. Although midwives are performing their duties since old times and are effectively managing the delivery processes of women; there are some constraints related to their limited knowledge that leads to unsatisfactory outcomes. For instance, residents pointed to the fact that midwives in Pakistan are mostly elderly women who are not much educated and do not have knowledge of critical patient management. They do more harm than good. For example, one resident stated,

“Midwives have opened the clinics in the periphery of the hospital. If they don’t know how to manage, then I always say please refer timely. They send the patient when everything from their hand has passed, and we have just 4–5% left. The midwife had badly injured the gut with the forceps that her (pregnant woman) uterus perforated inward, and the gut protrudes out

(p.9)

A participant talked about the cases handled by midwives. Midwives have opened their clinics in the periphery of the hospital. They treat pregnant women but have limited knowledge regarding complicated cases. Sometimes they cannot manage the complicated patient timely and refer the patient to tertiary care at the last moment when there are the least chances in patient wellness. This is the dilemma of our society that they trust midwives more than doctors. Furthermore, participant narrated a story of a woman who came with a ruptured uterus and was bleeding heavily. The doctors when opened the patient in OT, they came to know that not only her uterus was ruptured, but her gut was also injured. The doctor’s hand infused into the posterior walls of the stomach. This is the midwife mishandled case who should timely refer the patient to the doctor instead of injuring the patient’s body.

#### Language barrier

This subtheme is related to patients who come from far-off areas and have issues with communication. It is a growing problem in Pakistan where misinterpretation of patient complaints or presentations occurs because *Urdu* (The national language of Pakistan) is not the native language of patients. Many people involved in patient care including laboratory personnel, nursing staff, physicians and residents’ trainees feel the potential language barrier that may negatively impact any ongoing relationship between providers (residents) and patients as well as impact their medical care. For instance, one resident stated,

“We have a major issue when people from periphery areas like *Koshistan* come to us. They neither speak Urdu nor Pushto. They cannot give a proper history.”

(p,13).

**Fig 1:**
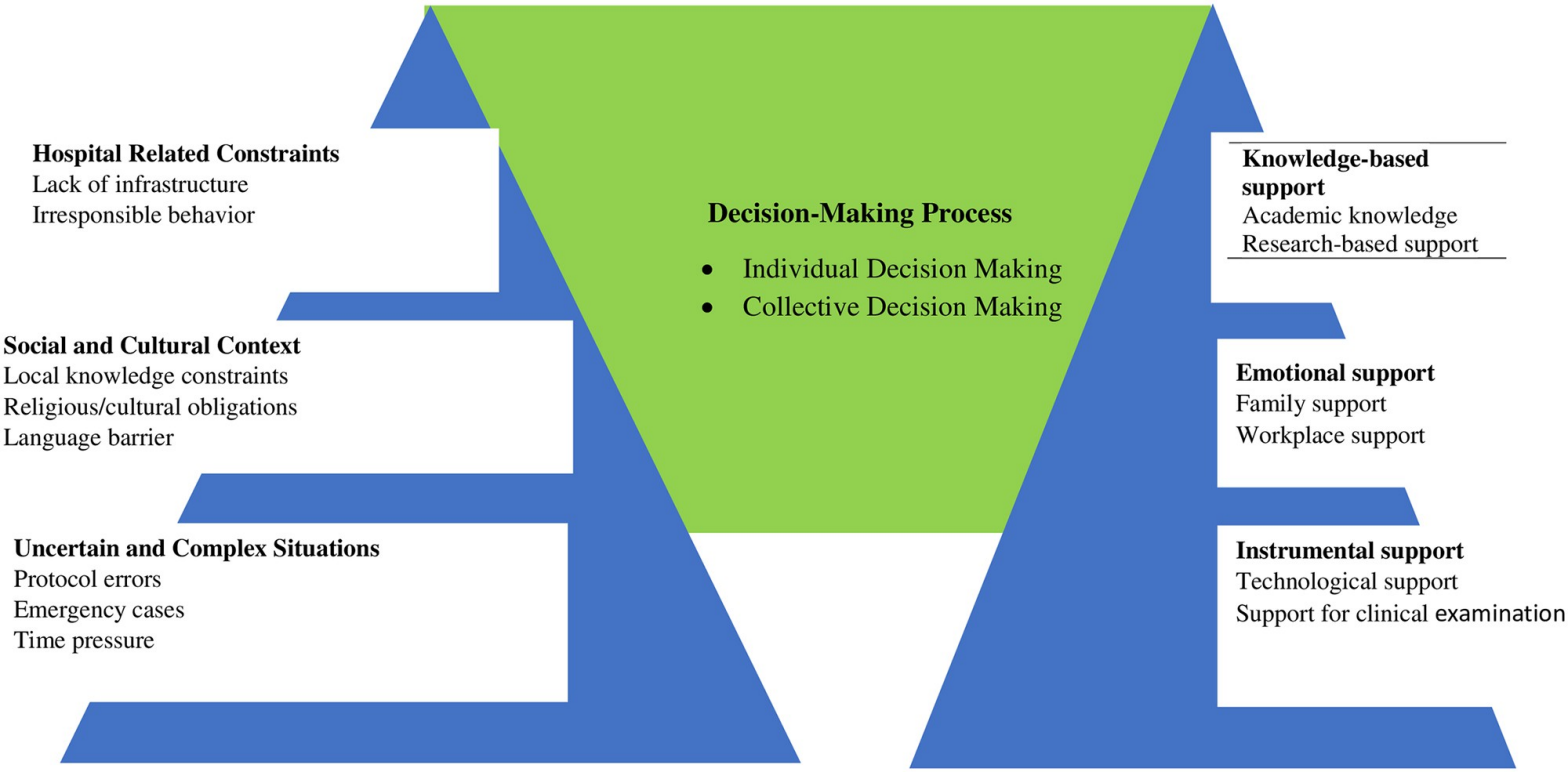
Challenges and support system in decision-making process.

This is the challenge associated with communication from people who cannot understand the national language of Pakistan (Urdu). People coming from periphery areas like *Kohistan* cannot understand *English*, *Urdu*, *Punjabi*, *or Pushto*. Not only do the patients and attendants feel difficulty in communicating with residents but also the residents have to put much effort to understand the patient’s history. There remains uncertainty in taking patients whether the medical reports and other measures have been checked or not, and how much labor pains the patient has taken. Since, the delivery time is highly dependent on these measures and any complications if come, lead to the mishandling of the patient. The overall picture of challenges in decision-making process and the support system that residents use is given in Fig 1.

## Support system in decision making

This is the second major category of the study. Residents use support in decision-making, divided into three themes: knowledge-based support, emotional support, and instrumental support. This is shown in Table 3.

**Table 3:** Support system in decision making.

Support System	
Knowledge-based support	Academic knowledge
	Research-based knowledge
Emotional support	Family support
	Workplace Support
Instrumental support	Technological support
	Support for clinical examination

### Knowledge-based support

Research, guidelines, and books are valid sources of information and support in decision-making. Informational support is available for residents in literature, medical guidelines, and books. It is further divided into two subthemes (i) Academic knowledge (ii) Research-based knowledge.

#### Academic knowledge

Academic knowledge is gained during five years of MBBS in medical school. Participants learned theoretical portions during academics that helped in understanding and increasing their knowledge of medical issues. Academics include the subjects related to eye, medicine, gynae, peads, etc. but only theoretical understanding is given in medical school. For example, one resident said,

“We are not given its training during MBBS. We people had the subjects, community medicine, gynae obs, pediatrics, behavioral sciences, eye; these subjects are taught to us but even in that…. task management was never told to us. We were never taught what problems will be faced by working in government setup. Also, patient-related communication was not taught to us.”

(p.13)

In this extract, a participant expressed their learning during MBBS. They were taught different subjects in each year like community medicine, gynae, pediatrics, eye, behavioral sciences etc. They have medical academic knowledge. They were given exposure to patients in hospitals but were not given practice or any training related particularly to decision-making in their academics. They were not taught the problems they would have to face in practical life as a young graduate trainee. Managing emergency patients or communicating with them was never taught in academics.

#### Research-based knowledge

Participants view that research-based knowledge is gained through evidence-based guidelines, research papers, and books but this type of exposure is gained during residency. Residents practically experience dealing with complex cases, management, and planning for treatment. Medical guidelines and books are sources of knowledge for them. For instance, one resident stated,

“Research, guidelines, and books; our madam has made the protocols. Our madam’s book has different chapters. All the protocols are briefly written in 10–15 pages. Okay….”

(p.9)

In this extract, participant elaborated on the support gained through research, books, and guidelines during residency. Their supervisor who is also the head of the department has authored books on gynae that help in gaining knowledge related to every disease, medicine, treatment plan, etc. The specialty of the supervisor’s book is that each chapter is given in a sequence consisting of an outline, and other necessary information in ten to fifteen pages.

### Emotional support

The emotional support was reported by participants at two levels, one from family members (family support) and the other from colleagues, seniors, and supervisors (workplace support).

#### Family support

The support provided by family members is emotional support as residents work thirty-six hours of hectic job and they only found relief with family members. The hospital is reported to be a continuous source of stress. For instance, a resident stated,

“To bear the stress level of gynae, this does not come from the department, it is from the family. If you have a good family system, you can do gynae. I will not say for any other (department). Only those girls who have passion….one cannot forcefully come in gynae.”

(p.5)

In this chosen extract, the participant detailed the support provided by family members. According to the participant, the support is only provided by parents, husbands, and understanding in-laws. Particularly, gynae is the most stressful department in the hospital as viewed by the participants. Hence, to bear the stress of gynae ward, only the ones having a supportive family, motivation, and passion can work. Otherwise, family problems, children, or other socio-cultural barriers like night calls create a strong barrier for women of gynae to pursue their careers.

#### Workplace support

Colleagues and senior residents provide workplace support and help in the learning and decision-making process of a patient’s management plan. When complex patients like eclampsia come with heavy vaginal bleeding, then junior residents see how the senior resident manage the emergency case and learn from their experience. Moreover, they engage in a discussion with supervisor who gives guidance in decision-making of critical patients. One resident reported,

“We assist the senior resident in cesarian from the first day of training. Then for a much longer time, we keep on assisting; afterward, we can do any task independently. As first-year trainees, we observe the seniors but afterward, we do it ourselves. If something seems too difficult, then we call senior.”

(p.3).

Participant expresses the support they gain from colleagues and seniors. During the initial years of residency, participants only assist in the cesarian delivery of a patient. They observe and learn through the experiences of seniors including their supervisors. For a long period, they keep on assisting in different medical tasks related to patient management. After they pass IMM (Inter-Mediate Module) exams and become third-year residents, then they are allowed to do any procedure independently as they have gained knowledge, experience, and learning. Even in that stage, if they feel difficulty in handling any case or they feel to discuss before decision-making, they call seniors (senior registrar or supervisor) for help.

### Instrumental support

At the organizational level, instrumental support is present. Instrumental support is available for residents to assist in patient diagnosis and assessment. This is a form of tangible support provided by the hospital in terms of machinery, laboratories, and other useful technology like advanced scanning. The instrumental support is divided into two forms (i) Technological support (ii) Support for clinical examination.

#### Technological support

Residents use ultrasound machines to assess fetal heart rate, fetal wight, the presence of liquor, and overall fetal growth. Liquor is the liquid that provides nutrients and oxygen to the fetus. Its quantity must be in optimum level; both the excess or minimization of liquor is harmful to the fetus. When there is a chance of human error in diagnosis or assessment then the machine may help in correctly detecting the problem. Residents use cardiotocography (CTG) machines to assess fetal hearts. Fetal distress can also be assessed through CTG machines.

“In the emergency room, we have a scan machine, also a CTG machine. CTG is essential before 15 minutes of shifting to OT.”

(p.10).

In this extract, participant elaborate on the usage of ultrasound scan machines and CTG machines which help examine fetal-related information. Before the cesarian, an assessment of fetal hearts is necessary that baby is fine and not going into distress.

#### Support for clinical examination

Residents view that they make decisions based on clinical examination of patients. But along with that clinical examination, they also use the support of medical devices and technology to validate their decisions. Although medical devices are a source of instrumental support, decisions are never made based on them. They can only make work easier but decisions are made on the doctor’s clinical examination. Sometimes, the medical device can give errors and faulty findings, hence a doctor is trained to examine, investigate, and treat the patients with their clinical knowledge.

“Technology and clinical go parallel. In gynae, you can know from the assessment. You have the patient fully dilated, you do not have time for an ultrasound or CTG scan. You can examine the baby’s movement by checking through your hand. No doubt, they have many roles. But you have to keep your clinical with this”.

(p.12)

In this extract, a participant explains the support for clinical examination. Doctors are trained to clinically examine the patient. Medical device always comes at a later stage. Doctors use their hands to detect the baby’s movement and position. Completely relying on ultrasound machines or CTG scan machine can make one dependent on medical devices which have the chance to give faulty readings. Although they do assist in clinical examination, hence both have an equal role in providing support for decision-making.

## Decision-making process

This is the third major category of the study. The decision-making process is influenced by the challenges faced by the residents at the individual as well as organizational level. The themes and subthemes are given below Table 4.

**Table 4:** Themes and subthemes related to the decision-making process.

Themes	Subthemes
Individual Decision making	Updated knowledge
	Rigorous vigilance
Collective Decision Making	In-house group decision making
	Multidisciplinary team decision making

### Individual decision making

In the settings of Obs and Gynae, individual decision-making takes place for non-critical tasks that do not require assistance from immediate seniors. For instance, the management of normal delivery and elective cesarian without any complications is included in non-critical tasks. These decisions can be taken by an individual senior resident, senior registrar, or even a consultant. Individual decision-making is further divided into two types given below.

#### Updated knowledge

The individual decision-making process includes updated knowledge regarding patient diagnosis and management. Residents keep themselves updated by reading the medical guidelines of the College of Physicians and Surgeons, Pakistan. Research papers also help in updating knowledge. Furthermore, learning gained through knowledge and experience in the ward of Obs and Gyne helps generate cues and patterns that help in decision-making. Recent empirical research helps in learning the etiology, prognosis, and treatment plan of gynae-related issues. One resident reported,

“Your knowledge of the patient’s diagnosis must be correct and the patient should be given good counseling that she agrees with your decision. The complications that can occur must be known to the attendants. Secondly, you have an alternative plan for any decision. Thirdly, you can follow the decision afterward, okay.”

(p.8).

In this selected extract, the participant elaborated in detail on the individual decision-making process. First is the knowledge regarding patient diagnosis. This knowledge is gained through evidence-based guidelines, research, books, and routine seminars including workshops on gynae-related issues. Second, good counseling of patients is necessary to give them awareness regarding pregnancy-related issues. The doctor should know the pros and cons of cesarian and other pregnancy-related complications like ectopic pregnancy. Attendants should be involved in decision-making. If the pregnancy is in the tube (ectopic pregnancy) then before the removal of the tube attendants must be counseled, and they should be told the harms of ectopic pregnancy for the patient. The third major thing is doctors should have an alternative plan option if things do not work well. Even if the doctor’s diagnosis is not correct then they should have an alternative plan that comes with knowledge and learning. The fourth thing is doctors must have the ability to take follow up on their decisions.

#### Rigorous vigilance

Rigorous vigilance involves two main types. The first is related to the time factor and the second is related to the commitment to action. In Obs and Gynae, timely decision is necessary. For instance, a patient having excessive bleeding needs timely management. Slight negligence can lead to serious consequences. The other factor is vigilance in committing action. The doctor should be vigilant to take quick action. An alternative treatment plan should be applied for patient safety if a certain management plan does not bring desirable results. One resident stated,

“The decision you are taking has not been delayed much.”

(p.5).

**Fig 2:**
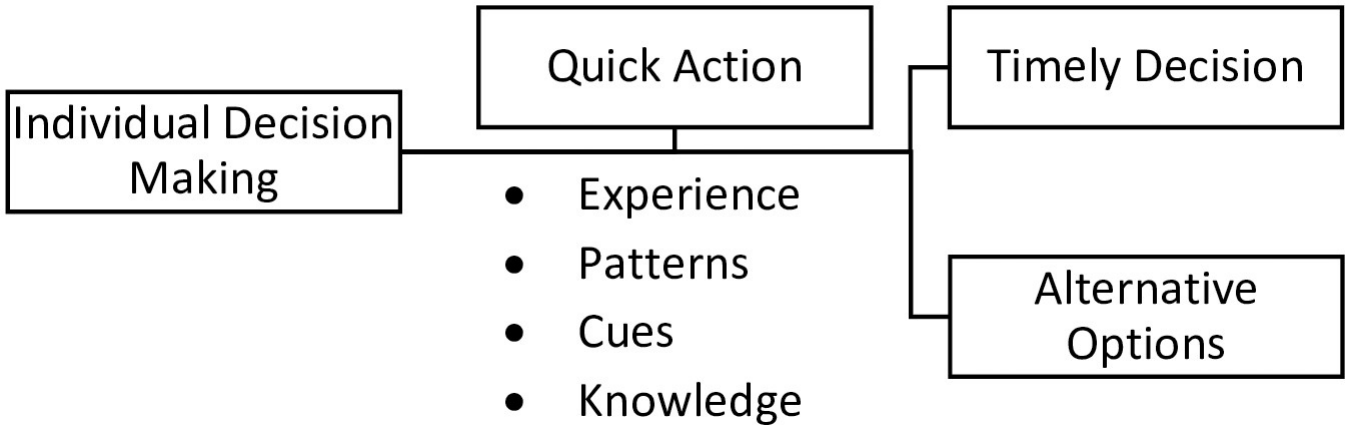
Individual decision-making process.

Here, the importance of timely decisions is reflected that they must not be delayed. This is only possible when the doctor has enough knowledge of diagnosis, examination, investigation, treatment, and follow-up. Individual decision-making is shown in Fig 2.

### Collective decision making

This type of decision-making occurs when residents discuss the case with seniors and then make decisions with their consent. This differs from individual decision-making in the manner that it is mostly involved with critical patients in an emergency. For instance, for complex cases, decisions are made with the consent of on-floor seniors, who discuss with the senior registrar, who further discusses with the Assistant Professor (AP). In this way, decision-making takes place in a chain process. Collective decision-making has been divided into two categories, i.e., (i) In-house group decision-making and (ii) multidisciplinary team decision-making.

#### In-house group decision making

This type of decision-making involves members of the Obs and Gynae ward. For instance, for complex cases, there are three main protocols. (i) The junior resident informs the senior who in turn informs their immediate seniors including senior registrar. This not only helps in decision-making but also gives experience to the junior residents regarding management of complex cases. In this way, they coordinate with each other and execute the action plan. For instance, one resident explained in these words,

“I will consult from my senior, PG2 (post-graduate of the second year) and PG 3 (post-graduate of the third year) they will consult from their seniors PG 4. The decision of PG 4 is right. If you think that decision should be discussed from above their level (4th year’s PG) then we have consultants. Above them are Professors. In this way, we discuss in a chain. We are taking decisions all the time and are consulting with seniors.”

(p.4).

**Fig 3:**
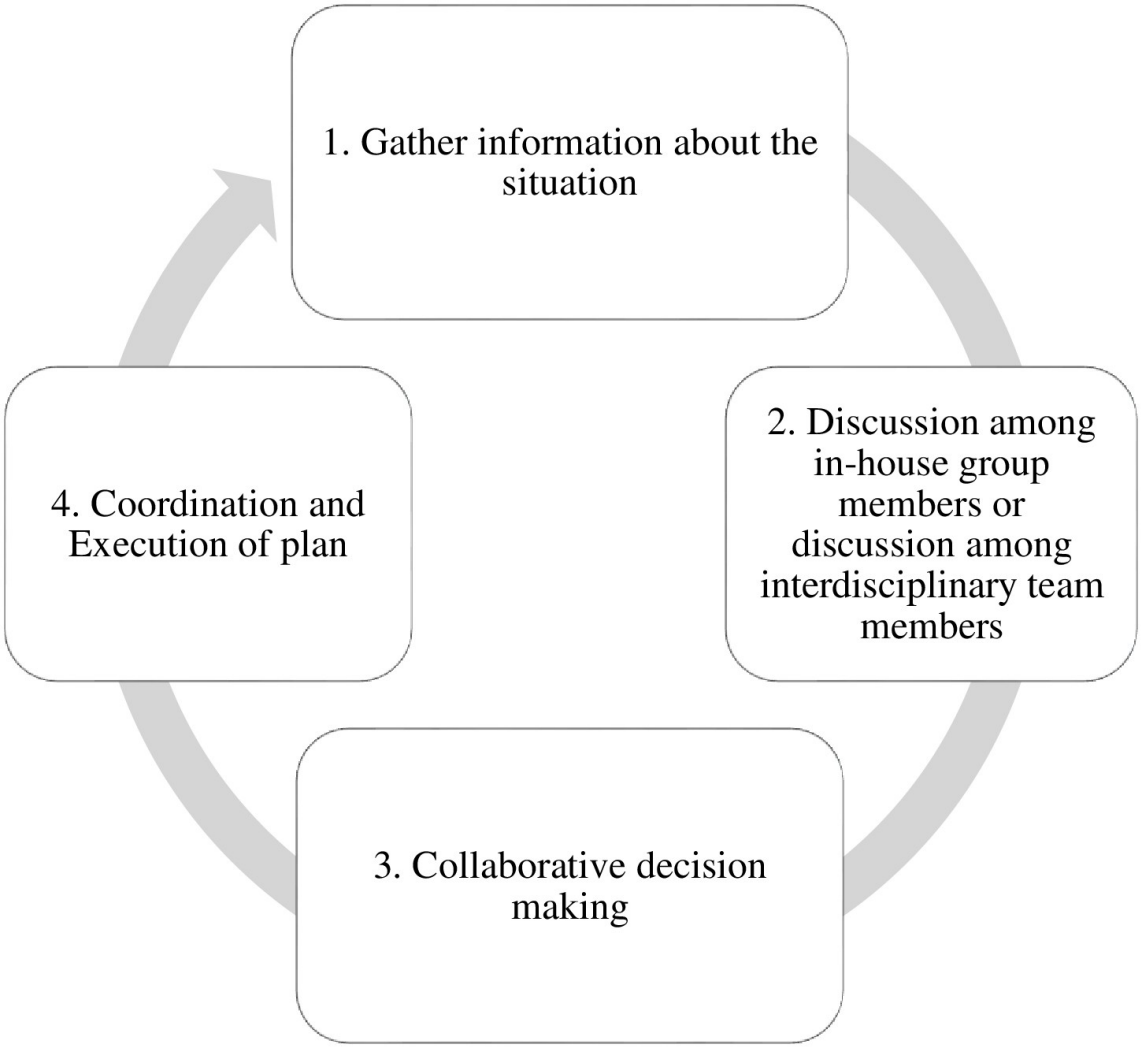
Collective decision-making process.

Here the resident explained the chain of commands. Junior residents of second and third year inform the senior resident of fourth year because they work in a group of 8–9 people consisting of junior residents, senior residents, the senior registrar, and a supervisor who is at the AP level. Above the APs are Professors. Any complicated case like hysterectomy is discussed with seniors, they discuss, collaborate with each other, and then decision is made. Fig 3 shows the collective decision-making process.

#### Multidisciplinary team decision-making

This is the second subtheme in collective decision-making process that describes the involvement of a multidisciplinary team in decision-making at the organizational level. This reflects the involvement of departments other than Obs and Gynae including Radiology, Medicine, Anesthesia, Blood Bank, and Pediatrics’.

“The decision of radiologist can be considered final. If residents do a scan, then two or three people cross-check before giving final decision. With radiology, with anaesthesia, yes mostly with these, and then with medicine, surgery, ICU settings also come with them.”

(p.9)

The radiologist can give his decision after scanning the baby through a detailed ultrasound. For instance, if a radiologist sees any anomaly in the scan, then he can report and his decision is considered final because of his specialty in radiology. Anesthesiologist is also specialized to give local or general anesthesia as per patient’s medical history and physical condition. The quantity of anesthesia can only be manipulated by the anesthesiologist as per the need of the patient. Then the surgery ward is specialized in doing surgeries of patients. Furthermore, the medical specialist can help in case the patient is handled by a midwife who used undue medicine that caused the infection. The ICU setting is a place where multiple medical and paramedical staff is available to assist in patient management. Obs and Gynae does not work in isolation rather need multidisciplinary teamwork for patient management.

## Discussion

As described in the beginning, the challenges of the Pakistani healthcare system and the decision-making process by practitioners including the residents seem to be marginalized. Lack of a uniform health insurance system, lack of trained staff, lack of access and unequal resources, poor governance, patient influx, and workload [24] create challenges in decision-making process. It is the biggest dilemma of the Pakistani health care system that doctors including residents show non-adherence to medical protocols in patient management and treatment. The errors can also lead to harsh consequences like patient death. However, due to complexity of the case, it is difficult to recognize the exact cost of compensations and liabilities on healthcare organizations, hospitals, and doctors [25]. Here, it is also reflected how doctors unknowingly make errors in decision-making, and sometimes in rare situations, trainees blindly believe the specialists without questioning.

The next major constraint is the emergency cases that doctors have to face. There is a huge overload of patients in tertiary care hospitals in Pakistan. Emergencies are coming at any time of day which includes patients with heavy bleeding, eclampsia patients, tubal ectopic pregnancy, and patients with fits. These emergency issues are related to time pressure as doctors have to immediately decide for patient further management. For instance, as revealed by the study participants the decision regarding eclampsia has to be taken timely otherwise patient may get shocks and brain injury. Here the shocks refer to fits (convulsions of brain) due to high blood pressure which increases the risk of brain damage. Similarly, there is a risk of uterine tube rupturing because of ectopic pregnancy. Hence, timely decisions and commitment to quick action are required by the residents. Here the patient is in a passive role but it is the practitioner who decides on behalf of the patient that what should be the accurate management plan for the critical patient. This view is in accordance with the technocratic model of birth [6] which views a pregnant woman as an object and a practitioner having the authority to provide care from outside.

Furthermore, the doctors perform maximum efforts in saving the life of the mother and child but there are some constraints of tertiary care hospitals. On one side, we operate our health care system like a big cottage industry with state-of-the-art technologies for patient care but the available infrastructure is inadequate. This includes a lack of adequate medical facilities including the non-providence of medical lab tests, non-availability of beds, instruments, and medical drugs. It has been reported that the public health care system faces many challenges in providing quality care to patients because of low budget allocations by the Government of Pakistan. The huge cost of private as well as public hospitals further drags vulnerable population groups into a poverty trap [26]. Research evidence also showed that Pakistan has a widespread infrastructure but inadequate resources i.e., technologies, supplies, and instruments [14].

The non-cooperation of paramedical staff and the non-availability of doctors at the time of emergency is an additional burden that influences decision-making. This becomes a constraint in decision-making as non-cooperation and irresponsible behavior of the staff is a potential threat and may lead to serious long-term consequences for patients. It has been estimated that the doctor-to-patient ratio in Pakistan is 1:1300 whereas WHO suggests that the doctor-to-patient ratio should be 1:1000 [17]. This is because Pakistan is the sixth most populated country in the world and the health resources as per the requirement of the population are scarce.

The sociocultural context also provides a challenge for Obs and Gynae practitioners. The role of midwives can never be disregarded in the management of pregnant women. In Pakistan, efforts are being done to empower the role of midwives to empower women. Currently, in Pakistan, some midwifery cadres are having different levels of education and provide child and maternal health care services [27]. On the other hand, the practitioners view the role of midwives as doing more harm to patients than good. For instance, lack of adequate knowledge regarding the treatment of critical pregnancy cases and usage of undue medicine further harms the patient that they do not have to take only long-term antibiotics but also suffer from serious consequences like hysterectomy (removal of the uterus). Unqualified practitioners are considered to be the sole reason for providing 50% of the health care services to the community population including urban and rural areas as reported in several studies [28]

The observance of cultural practices by patients becomes a hurdle in doctors’ decision-making. For instance, patients have a lack of awareness regarding the usage of contraception considering it a sin, and associate it with infertility, allergies, and irregularity in menses [29]. Sometimes it is associated with a symbol of shame and guilt. Our society’s dilemma is that they talk about all the topics openly but the topic of pregnancy-related concerns is given the least priority considering it a taboo topic. When residents try to educate the patients and their attendants regarding the effective usage of contraception and its benefits for patient’s health, they show reluctance to use it and do not get counseled. On the other hand, some patients coming from the periphery areas have issues communicating with the doctors. They cannot understand and speak the local languages of Pakistan and understand only their rural dialects. Doctors feel difficulty in taking history of such patients and making effective decision plan for their management. Here, it is the dilemma of our system that our health care system does not provide training to doctors for effective communication and informed decision-making. However, evidence shows that patients also show anxiety with their inability to communicate and participate in decision-making. Many patients, however, have expressed frustration with their inability to participate in decision-making and in systems of care responsible for their family’s values and needs [30].

With a lack of infrastructure, protocol errors in patient care, dealing with time pressure, emergency issues, and sociocultural barriers, it can be concluded that doctors have to face challenges at every level. Not only this, but the five years of medical training in MBBS also do not teach how to make effective decisions. Additionally, residents observe the seniors’ actions and gain experience in critical task management for learning. The gained experience helps in building pattern recognition. Our system has a constraint that it does not teach residents the art of effective decision making which is essential in patient management and care. It can be argued that individual decision-making is learned by updating self-knowledge, observation, research, and guidelines but the actual confidence to make decisions in times of uncertainty is still not learned by residents as the system does not allow them to take independent decisions.

Residents make collective decisions through discussions with seniors including consultants regarding complex case management (for instance, a complex case of ectopic pregnancy). The in-house group decision-making allows them to learn, discuss, collaborate, coordinate and execute the plan. In collective decision-making, a hierarchy is followed in which physicians dominate and the system fails to solicit the contributions of those residents who have added their insight and relevant information for patient management [31]. Moreover, the multidisciplinary group decision-making looks towards the combined efforts of all departments including Obs and Gynae, radiology, medicine, surgery, and anesthesia in decision-making. The input from multiple individuals can be more accurate than one expert because of collective intelligence [32]. With decision-making at different levels and the existence of constraints, residents also have a support system to use in decision-making.

Guidelines, research evidence, and medical books provide a source of informational support to the residents. Similarly, the emotional support provided by family members as well as colleagues is an additional protective factor that may play a positive role in residents’ work engagement as well as decision-making. Team support and effective communication among colleagues are important factors for team members in order to coordinate as well as to adapt to the changing dynamic environment of the health care system. Research evidence also revealed that teamwork is involved in every task including the tasks performed in the operation theater, emergency, and Intensive Care Unit (ICU) [33]. Additionally, the role of medical devices and technology including ultrasounds machines, and CTG machines cannot be neglected with the clinical examination in decision-making.

Internationally, research is available on decision-making in other departments like oncology, where decision-making is not solely based on evidence-based medicine rather eminence-based decision-making where the opinion of an experienced supervisor comes into play. Decision-making in oncology is categorized into three categories. The contextual factors include influence from the pharmaceutical industry, treatment costs, healthcare systems, and patients’ socioeconomic status. The decision-maker-related characteristics include both the physician and the patient. These factors include attributes such as degree of expertise, frames of reference, emotions, self-efficacy, and confidence. And the decision-specific criteria which includes classical clinical criteria, such as expected treatment toxicity, biomarkers, presence of comorbidities, age, or performance status [34]. Hence, even in departments other than Obs and Gynae, multiple factors influence the decision-making of physicians which cannot be ignored. Generally, decision-making in medicine encompasses all health-related contexts by patients, the general public, health policymakers, and healthcare workers. Medical decision-making compares descriptive or actual decision-making to rational or normative models of decision-making. For instance, in a recent study, the authors developed ADAPT, a mnemonic framework to improve medical students’ and surgical residents’ comprehension and recall of important steps in uncertainty disclosure which is pervasive in medicine from sensitivity of diagnostic tests and differential diagnoses to the absence of a single path of recovery for the patient. This helped residents’ in the assessment of patients’ temper expectations, acknowledging patients’ emotions, knowledge, disclosure of uncertainty, and planning the next steps [35].

As decision-making in Obs and Gynae and other healthcare specialties is also taking into account contextual as well as decision-task factors hence it is also interesting to discuss the healthcare system of other countries such as United States (US). We found that a well-trained health workforce and a large number of high-quality medical specialists, a robust health sector research program as well as secondary and tertiary institutions with the best medical outcomes are found in this region. But at the same time, it also suffers from unequal distribution of outcomes and resources among different population groups, poor measures on many subjective and objective measures of quality, health expenditure levels per person far exceeding all other countries, and incomplete coverage of its citizenry [36]. Pakistani healthcare system including physicians of Obs and Gynae can learn from US health system to empower their primary and secondary care centers with well-equipped instruments and specialized doctors, only then the high patient influx of tertiary care could be minimized as well as doctors’ workload will decrease. This will not only decrease the chances of errors in medical decision-making but will also increase patient satisfaction.

## Conclusion

The study explores the Obs and Gynae residents’ challenges of decision-making, decision-making process, and support usage across Pakistani tertiary care hospitals. Numerous challenges have been reported by the residents including task-related (uncertain and complex situations, time pressure, and emergency issues), infrastructure challenges, irresponsible behavior of staff, and socio-cultural contextual challenges. Individual and collective decision-making both take place with the help of evidence-based, informational, experience-based, and research-guided knowledge. The knowledge-based, emotional as well as instrumental support is found to be the protective factor in decision-making. The study also brings up a thought-provoking question; the way our health care system prepares young trainees without educating them about decision-making and what they are called upon to do in practice, these gaps are attributable to many factors including lack of adequate funding for curriculum revamp.

### Implications

Professionals, decision-making researchers, and practitioners (of not only Obs and Gynae but also from other departments) interested to study the challenges of decision-making in complex and dynamic settings of health care can find this study as a useful source based on empirical data. Additionally, this study is a useful addition to the literature on health care, health policy, decision-making frameworks, cognitive psychology, obstetrics and gynecology, and medical studies. Hence, the study provided a transdisciplinary approach to study decision-making for researchers as well as practitioners in hospitals.

The precise lessons that can be given to young physicians in Obs and Gynae in the light of the present study are physicians should put their learning and experience together while making decisions, they must follow their seniors but at the same time, they should also question the medical judgments of seniors. This would help them to make better decisions and improve their learning. Blindly following seniors may lead to poor consequences. Moreover, physicians should be critical in analyzing any medical diagnosis. If it leads to grave consequences, they must have an alternative treatment plan. This is only possible with research-based updated knowledge as well as vigilance in taking action. Hence, trainee residents should keep themselves updated regarding new procedures, guidelines, and techniques of Obs and gyne. Additionally, regarding patients and their attendants, young physicians should try to understand and empathize with the patients, involve them in decision-making, and counsel them in every matter related to pregnancy, its complications, family planning, and general maternity advice considering it part of their duty. This will not only provide benefits to the patients but also improve the medical counseling skills of physicians.

## Supporting information

S1 Appendix Consisting of interview guideline questions. (DOCX)

S2 Appendix Consisting of dataset. (XLSX)
