# Supplementary material for: Decision making among residents in training of obstetrics and gynecology: A qualitative exploration in Pakistani context
Source: PLoS One. 2023 Nov 2;18(11):e0287592. doi: 10.1371/journal.pone.0287592 (PMC10621809; doi:10.1371/journal.pone.0287592)
Supplement: S1 Appendix — (DOCX) [file pone.0287592.s001.docx]

**Interview guideline questions**

1. Do you make decisions in your departmental setting?
2. What type of decisions do you make? Please elaborate.
3. Do you face complex tasks in your departmental setting?
4. What do you mean by task complexity in your departmental setting?
5. Do the five years of MBBS curriculum involve teaching decision-making of complex tasks?

**Probe**: As a resident, are you taught decision-making about complex tasks? Please elaborate.

1. Do you face uncertain situations in your departmental settings?
2. Do you make decisions in an uncertain situation?
3. How do you perceive decision-making under uncertain situations?
4. How do you perceive decision-making under stress?
5. Do you have any support system to use in decision-making under uncertain situations?
6. What types of support systems do you have in your departmental setting that help in decision-making? Elaborate

**Probe:**

1. Can you give an example of a computer-based support system that you have used in your departmental setting?
2. What are the specific features of that support system?
3. How do you perceive the support system in terms of usage and misusage?
4. Do you make decisions under time pressure?
5. What type of tasks involves time pressure?
6. How do you perceive decision-making under time pressure?
7. Do you think any factors related to the hospital create any stress in decision-making? If yes, how?
8. How does hospital culture influence decision-making?
9. Do you face hospital-related constraints in your departmental settings?
10. How do you perceive decision-making under hospital constraints?
11. Do you face any pressure from the people in your social surroundings when you make decisions?
12. Do you face pressure from the patient or patient's family while decision-making? Please elaborate.

**End of Interview Close**

“Thank you for your time. Is there anything you wanted to discuss that I missed or did not cover? Did you want to add anything else to any of your responses?”

“If there are any further questions, comments, or concerns do not hesitate to contact me. My information is on your consent form.”
